# Mitragynine Attenuates Morphine Withdrawal Effects in Rats—A Comparison With Methadone and Buprenorphine

**DOI:** 10.3389/fpsyt.2020.00411

**Published:** 2020-05-07

**Authors:** Rahimah Hassan, Cheah Pike See, Sasidharan Sreenivasan, Sharif M. Mansor, Christian P. Müller, Zurina Hassan

**Affiliations:** ^1^Centre for Drug Research, Universiti Sains Malaysia, Minden, Malaysia; ^2^Department of Human Anatomy, Faculty of Medicine and Health Sciences, University Putra Malaysia, Serdang, Malaysia; ^3^Institute for Research in Molecular Medicine, Universiti Sains Malaysia, Minden, Malaysia; ^4^Section of Addiction Medicine, Department of Psychiatry and Psychotherapy, University Clinic, Friedrich-Alexander-University Erlangen-Nuremberg, Erlangen, Germany; ^5^Addiction Behaviour and Neuroplasticity Laboratory, National Neuroscience Institute, Singapore, Singapore

**Keywords:** mitragynine, kratom, morphine, withdrawal, substitution, methadone, buprenorphineI

## Abstract

**Background:**

Opiate addiction is a major health problem in many countries. A crucial component of the medical treatment is the management of highly aversive opiate withdrawal signs, which may otherwise lead to resumption of drug taking. In a medication-assisted treatment (MAT), methadone and buprenorphine have been implemented as substitution drugs. Despite MAT effectiveness, there are still limitations and side effects of using methadone and buprenorphine. Thus, other alternative therapies with less side effects, overdosing, and co-morbidities are desired. One of the potential pharmacotherapies may involve kratom's major indole alkaloid, mitragynine, since kratom (*Mitragyna speciosa* Korth.) preparations have been reported to alleviate opiate withdrawal signs in self-treatment in Malaysian opiate addicts.

**Methods:**

Based on the morphine withdrawal model, rats were morphine treated with increasing doses from 10 to 50 mg/kg twice daily over a period of 6 days. The treatment was discontinued on day 7 in order to induce a spontaneous morphine abstinence. The withdrawal signs were measured daily after 24 h of the last morphine administration over a period of 28 abstinence days. In rats that developed withdrawal signs, a drug replacement treatment was given using mitragynine, methadone, or buprenorphine and the global withdrawal score was evaluated.

**Results:**

The morphine withdrawal model induced profound withdrawal signs for 16 days. Mitragynine (5–30 mg/kg; i.p.) was able to attenuate acute withdrawal signs in morphine dependent rats. On the other hand, smaller doses of methadone (0.5–2 mg/kg; i.p.) and buprenorphine (0.4–1.6 mg/kg; i.p.) were necessary to mitigate these effects.

**Conclusions:**

These data suggest that mitragynine may be a potential drug candidate for opiate withdrawal treatment.

## Introduction

Abuse and addiction to opioids including prescription pain relievers, heroin, and synthetic opioids such as fentanyl caused a serious national crisis in the United States (US), that affects public health as well as social and economic welfare (1). According to Centers for Disease Control and Prevention (CDC), the opioid crisis has occurred in three waves. The first wave began in the 1990s with the rises of death cases related to overdose of prescription opioids. The second wave emerged in 2010 involving heroin overdoses. The third wave began in 2013 with the sharp rise of overdose death numbers involving synthetic opioids, particularly illicitly manufactured fentanyl (IMF). Fentanyl is 50–100 times more potent than morphine as an analgesic agent. In the US alone, more than 47,000 opioid-related overdose deaths occurred during 2017, which are closely related to synthetic opioids, especially fentanyl (2). At present, IMF still contributes greatly to overdose fatalities (3) in many states of the US and in Canada (4, 5). IMF fatalities are generally due to co-administration of other illicit drugs, such as cocaine, heroin, and methamphetamine (6, 7), which leads to overdose and consequently death.

As stated in the *World Drug Report 2019*, an estimated 271 million people worldwide used drugs and almost 13% are estimated to suffer from drug use disorder, to a point where they may experience dependence and/or require treatment (8). In Malaysia, opioid remains the main type of illicit substance, with estimated 187 771 opiate users. Mu-opioid receptor agonists are the mainstay in drug replacement therapy. Buprenorphine and methadone are recommended in pharmacotherapy of opioid use disorder based on their mechanisms of action in alleviating withdrawal signs (9). However, both have clinical limitations in their effectiveness and safety.

Typically, methadone therapy is considered as safe. It has a long half-life and makes outpatient management feasible. Nevertheless, several risk factors have been recognized, such as; i) drug–drug interaction with other drugs, ii) torsade de pointes, which elevated risk of some individuals, and iii) the inadequate or erroneous dose increase adjustment, specifically, when prescribing methadone for pain (10). Moreover, methadone efficacy shows high inter-individual variance due to pharmacokinetic and pharmacodynamic factors (11). The effective half-life of methadone for analgesia in many patients does not reflect the half-life for respiratory depression and cardiac side effects, making consistent and safe dosing difficult for methadone (12). This often leads to an overdose which is associated with serious side effects, including potentially lethal respiratory depression. Indeed, unintentional deaths are much more common after methadone administration than after any other opioid (13). Methadone fatality occurred not only among out-treatment patient but also for in-treatment patients. Fatality that occurred might be due to the methadone itself or due to drug–drug interactions. Caplehorn and Drummer identified 13 fatalities in methadone maintenance programs with 11 out of 13 cases related to methadone toxicity, one case of other drug toxicity and one case of other death causes in the first week. In the second week, 25 fatalities were reported with one case of methadone toxicity, 5 cases of other drug toxicity, and 19 cases of other death causes (14). Meanwhile, another study done by Kelty et al. demonstrated a high rate of mortality in methadone-treated patients during the first 28 days of treatment as compared to naltrexone-treated patients (15). Moreover, a study done by Bell et al. revealed 60 sudden deaths associated with methadone, where 19 out of 32 in-treatment cases and 24 out of 28 cases of out-treatment were linked to overdose (16). The autopsy results for all 43 methadone overdose positive deaths revealed at least one other drug was involved (16).

An alternative option to methadone is the buprenorphine replacement therapy. It acts as mu-partial agonist at mu opiate receptor (MOR) and has “ceiling effect” for respiratory depression. Thus, it offers advantages in terms of safety as compared to methadone. Buprenorphine has been approved in several countries as an efficient and safe maintenance therapy for heroin addiction which resulted in a salutary effect with a reduction in heroin overdose-related deaths in countries that implemented office-based buprenorphine maintenance. In France, however, several cases of asphyxia deaths were reported among addicts treated with buprenorphine concomitant with other drugs like benzodiazepines. Drug–drug interactions with buprenorphine may cause severe respiratory depression (17). Buprenorphine has been reported to cause seven fatality cases, four cases were associated with buprenorphine poisoning, followed by two suicidal cases and one undetermined case (18).

The kratom plant (*Mitragyna speciosa* Korth.) has been long used traditionally for its pharmacological effects and its narcotic action in Southeast Asian nations, especially Thailand and Malaysia. People use kratom plant preparations for their medicinal value in treating pain, mood swing, coughing, diarrhea, and intestinal infection (19, 20). Kratom is also used as a substitution of heroin and morphine when access to these drugs is prevented and as treatment for drug withdrawal signs (21, 22). Kratom leaves consist of over 25 alkaloids, where mitragynine is the main indole alkaloid (23).

Recent fatality reports by CDC revealed 152 deaths between July 2016 and December 2017, where multiple drugs were detected in almost every kratom-related death (24). Another study reported 156 death cases linked to kratom use, where 87% cases associated with polydrug use. Another 23% cases was due to kratom itself, with 6 cases found mitragynine in toxicology test (25). However, the claim that six fatalities was due to mitragynine alone does not mention the sources, thus, the truth on the claim was unclear. High post-mortem mitragynine concentration may not always reflect the direct involvement in a fatality (26). Gershman et al. reviewed the Colorado death certificates for any mentioning of kratom or mitragynine from 1999 through 2017. They found four death cases due to mitragynine. Further investigation on these four cases were performed through toxicology screening with high-performance liquid chromatography with tandem mass spectrometry using available residual blood. Result revealed that all these three death cases was indeed due to drug–drug interaction between mitragynine and other drugs instead of mitragynine compound alone (27). Death resulting solely from ingestion of kratom appears as extremely rare (28).

Mitragynine acts as partial agonist on human μ opioid receptor (MOR) and δ opioid receptor (DOR) while it acts as a competitive antagonist on human κ opioid receptor (KOR) (29). In addition, *in vivo* test showed that antinociceptive effects of mitragynine are mediated by supraspinal MOR and DOR (30). Boyer et al. reported that although mitragynine works as an agonist at the MOR, respiratory depression, coma, pulmonary edema, and death have not been associated with human kratom ingestion (31). Mitragynine, therefore, may exert several pharmacological effects that could help attenuating opioid withdrawal signs. An *in vitro* study from Kruegel et al. reported that mitragynine is a G-protein-biased agonist of the MOR, which does not recruit ß-arrestin following receptor activation (29). In particular, some evidence indicates that those MOR agonists that are biased toward G protein signalling over ß-arrestin signalling induce less respiratory depression, tolerance development, and constipation, while remaining potent analgesics (32, 33). In the present study we tested the effectiveness of mitragynine to mitigate morphine withdrawal behavior in rats, in comparison with the established substitution drugs, methadone and buprenorphine. We further evaluated the effects of mitragynine on morphine-withdrawal induced changes in hematological, biochemical, and histopathological parameters.

## Materials and Methods

### Animals

Male Sprague-Dawley rats were purchased from Animal Research and Service Centre, Universiti Sains Malaysia, Penang, Malaysia, weighing 200–300 g at the beginning of the experiment. All 84 rats were naive and used in a single experiment only. They were socially housed in groups of six per cage under standard laboratory conditions, with temperature- controlled environment (24 ± 1°C). The room was maintained on a 12-h light/12-h dark normal cycle (lights on from 07:00 to 19:00 h). Animals were handled for one week prior to commencement of the experiments. Food and water were available *ad libitum*. The experimental procedures were reviewed and approved by the Animal Ethics Committee of Universiti Sains Malaysia [Reference number: USM/Animal Ethics Approval/2016/(716)].

### Drug Preparation

Morphine hydrochloride, methadone hydrochloride, and buprenorphine hydrochloride were purchased from Sigma Chemicals Co. (USA). Mitragynine was extracted, isolated, and verified from fresh leaves of *M. speciosa* at the Centre for Drug Research, Universiti Sains Malaysia as described previously (34). Purified mitragynine was confirmed by high-performance liquid chromatography (HPLC) and proton nuclear magnetic resonance (1H-NMR) (400 MHz) analysis (35). Mitragynine obtained by this procedure was approximately 98% pure (36). Mitragynine was dissolved in 20% of Tween 80 as vehicle. Fresh stocks of morphine, methadone, buprenorphine, and mitragynine were prepared daily according to the weight of animals in the experimental design. They were dissolved in vehicle (20% Tween 80; Sigma Aldrich, UK) and injected intraperitoneally (i.p.).

### Experimental Design

#### Experiment I: Morphine Withdrawal Model

In this experiment, a morphine withdrawal model was developed by measuring the severity and duration of withdrawal signs. Morphine was injected with the dose that was progressively increased from 10 to 50 mg/kg twice daily over a period of 6 days (**Supplementary Table 1**). The vehicle group received 20% Tween 80 twice daily for 6 days. Both treatments were disrupted on day 7 in order to induce a spontaneous opiate abstinence. The withdrawal syndrome was evaluated daily over a period of 28 days after the last dose of drug treatment.

#### Experiment II: Mitragynine Substitution Treatment in Morphine Withdrawn Rats

Based on the above findings, the severity of spontaneous morphine abstinence was first observed, 24 h after the last dose of morphine. Hence, the substitutive treatment of drugs, i.e. mitragynine, methadone, and buprenorphine were administered before 24 h to prevent the emergences of morphine withdrawal syndrome. Selection of doses of mitragynine was based on previous studies (37–43). Mitragynine (0, 5, 10, 15, 30 mg/kg) was injected 30 min before withdrawal testing, i.e. 23.5 *h* after the last morphine dose (44, 45). Thereafter, mitragynine was repeatedly administered every 12 h (46) for testing the duration of withdrawal inhibiting effects. Treatment with mitragynine was then disrupted on day 5 in order to evaluate whether withdrawal signs would resurface after abrupt cessation. The withdrawal syndrome was evaluated 12 h after the last dose of mitragynine.

#### Experiment III: Methadone Substitution Treatment in Morphine Withdrawn Rats

The selection of methadone doses was based on pharmacological range and below LD_50_ value which might be effective and safe for the use in this experiment (47, 48). Methadone (0, 0.5, 1.0, 2.0 mg/kg was dissolved in vehicle (20% Tween 80; Sigma Aldrich, UK) and injected intraperitoneally 10 min before withdrawal testing, i.e. 23 h 50 min after the last morphine dose (47). Thereafter, methadone was repeatedly administered every 8 h (47) for testing the duration of withdrawal inhibiting effects. This design is a modified version of a substitution routine described by Ruiz et al., 1996 (49).

#### Experiment IV: Buprenorphine Substitution Treatment in Morphine Withdrawn Rats

The selection of buprenorphine doses was based on the previous studies (50–52). Buprenorphine (0, 0.4, 0.8, 1.6 mg/kg, i.p.) was dissolved in vehicle (20% Tween 80; Sigma Aldrich, UK) and injected intraperitoneally 30 min before withdrawal testing, i.e. 23.5 h after the last morphine dose (52). Thereafter, buprenorphine was repeatedly administered every 12 h (53) for testing the duration of withdrawal inhibiting effects. On day 5, buprenorphine treatment was interrupted in order to measure whether withdrawal signs would resurface after abrupt cessation. The withdrawal signs were evaluated 12 h after the last dose of buprenorphine.

### Assessment of Withdrawal Behaviors

Trained observers (RH and ZH, inter-rater reliability, r = 0.99) blinded to treatment and time points scored all images using the video and counted the frequency of the signs of spontaneous morphine withdrawal (chewing, head shakes, exploring, digging, yawning, teeth chattering, wet dog shakes, writhing, squeaking on touch, hostility on handling, diarrhea). Recording was conducted 24 h after the last dose of morphine administration. Animals were placed in an open field test box for 30 min and withdrawal behavior was scored. This test was performed on either day 28 (experiment I) or day 5 (experiment II–IV). The assessment of spontaneous opiate abstinence was performed by behavioral scoring. Withdrawal behaviors were distinguished as “counted signs,” including chewing, head shakes, exploring, digging, yawning, teeth chattering, wet dog shakes, writhing and as “checked signs,” including squeaking on touch, hostility on handling, and diarrhea. Thereby, counted signs and checked signs were further processed by multiplying with the respective weighing factors for evaluation of the severity of withdrawal signs using the previously described scoring methods by Rahman et al. (54), Bläsig et al. (55), Neal and Sparber (56), and Sabetghadam et al. (57) (**Supplementary Table 2**).

### Hematological Analysis

At the end of substituted studies, blood samples were collected *via* cardiac puncture and transferred into ethylenediamine tetraacetic acid (EDTA) tubes. Then, the tubes were analyzed for determination of hematological parameters such as red blood cell count (total RBC), hemoglobin, percentage of packed cell volume (PCV%), mean corpuscular volume (MCV), mean corpuscular hemoglobin (MCH), mean corpuscular hemoglobin concentration (MCHC), percentage of red cell distribution width (RDW%), total of white blood cell count (WBC), percentage of lymphocyte, monocytes, eosinophils, basophils, and platelet counts (PLT).

### Biochemical Analysis

For the biochemical analysis, the collected blood was transferred into serum-separating tubes. The biochemical parameters analyzed from these tubes were total bilirubin, aspartate amino transferase (AST), alanine aminotransferase (ALT), alkaline phosphatase, sodium, potassium, chloride, urea, creatinine, total cholesterol, triglycerides, calcium, phosphorus, total protein, albumin, globulin, and albumin/globulin ratio (A/G ratio).

### Histopathological Analysis

On day 5, animal tissue samples of targeted organs (heart, lung, kidney, liver) were harvested after behavioral testing in experiments II-IV. The tissues were stained using hematoxylin and eosin. Then, the slides were viewed under light microscope equipped with a digital camera. The sections were analyzed for structural changes, degenerative alterations, necrosis, and signs of inflammation.

### Statistical Analysis

All data were expressed as mean ± standard error of the mean (SEM). The individual behaviors scores, the global withdrawal scores, and the substitution treatments were analyzed by two-way ANOVA for repeated measures with test “day” as within factor and treatment combination as a “treatment” between factor. In order to analyze single group differences on each treatment day, pre-planned comparisons were calculated using Bonferroni test (58). Hematological and biochemical were also analyzed by two-way repeated measures ANOVA and Bonferroni test. A significance level of p < 0.05 was used to test for statistical significance. GraphPad Prism 6.0 software (GraphPad Software Inc., La Jolla, CA, USA) was used to perform the statistics.

## Results

### Global Withdrawal Scores After Spontaneous Morphine Withdrawal

**Figure 1:**
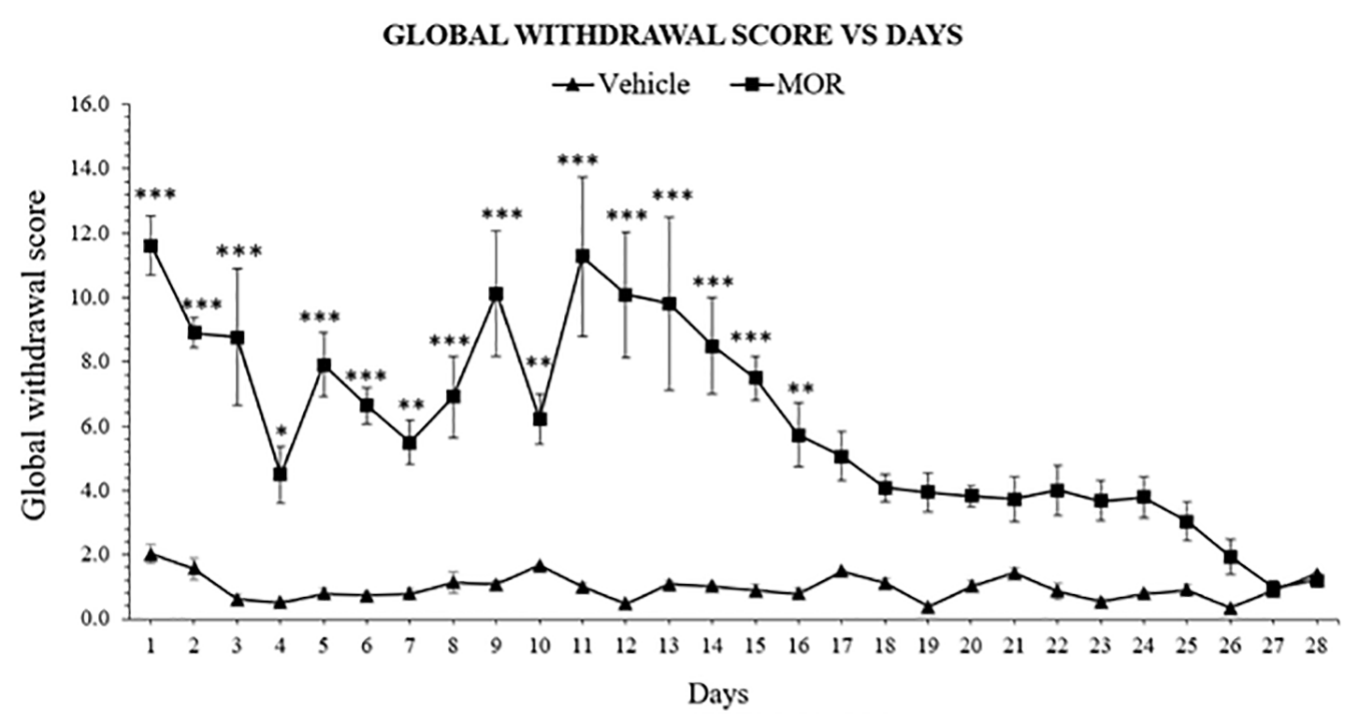
Escalating morphine (MOR) treatment induces withdrawal signs in rats. Data represent means (± SEM) of global withdrawal signs (n = 6/group; *p < 0.05, **p < 0.01, ***p < 0.001 vs. Vehicle).

The withdrawal model was adopted and modified from the dependence model of Rahman et al. (54). Single behavior scores were then calculated and translated into global withdrawal score to reduce variability and improve the reliability of results of morphine dependence (**Supplementary Table 3**) (59). Following abstinence, morphine dependent rats showed significant withdrawal signs 24 h after the last drug dose, with 11.614 ± 0.912 of global withdrawal score for the morphine group and 2.023 ± 0.291 in the vehicle group. Two-way ANOVA result showed significant effect of treatment (F_1,__168_ = 509.0, p < 0.0001), days (F_27,__168_ = 7.000, p < 0.0001), and an effect of the interaction (F_27,__168_ = 6.121, p < 0.0001). Bonferroni's multiple comparisons test revealed a significant difference of withdrawal scores between MOR-treated and vehicle control animals, which were observed from day 1 to day 16 (p < 0.05), and no statistical differences between day 17 and 28 (p > 0.05; **Figure 1**). Results indicated that behavioral signs of morphine withdrawal emerged after 24 h and might alleviated without any replacement drug treatment after 16 days abstinent period.

### Mitragynine Attenuates Morphine-Withdrawal Behavior

**Figure 2:**
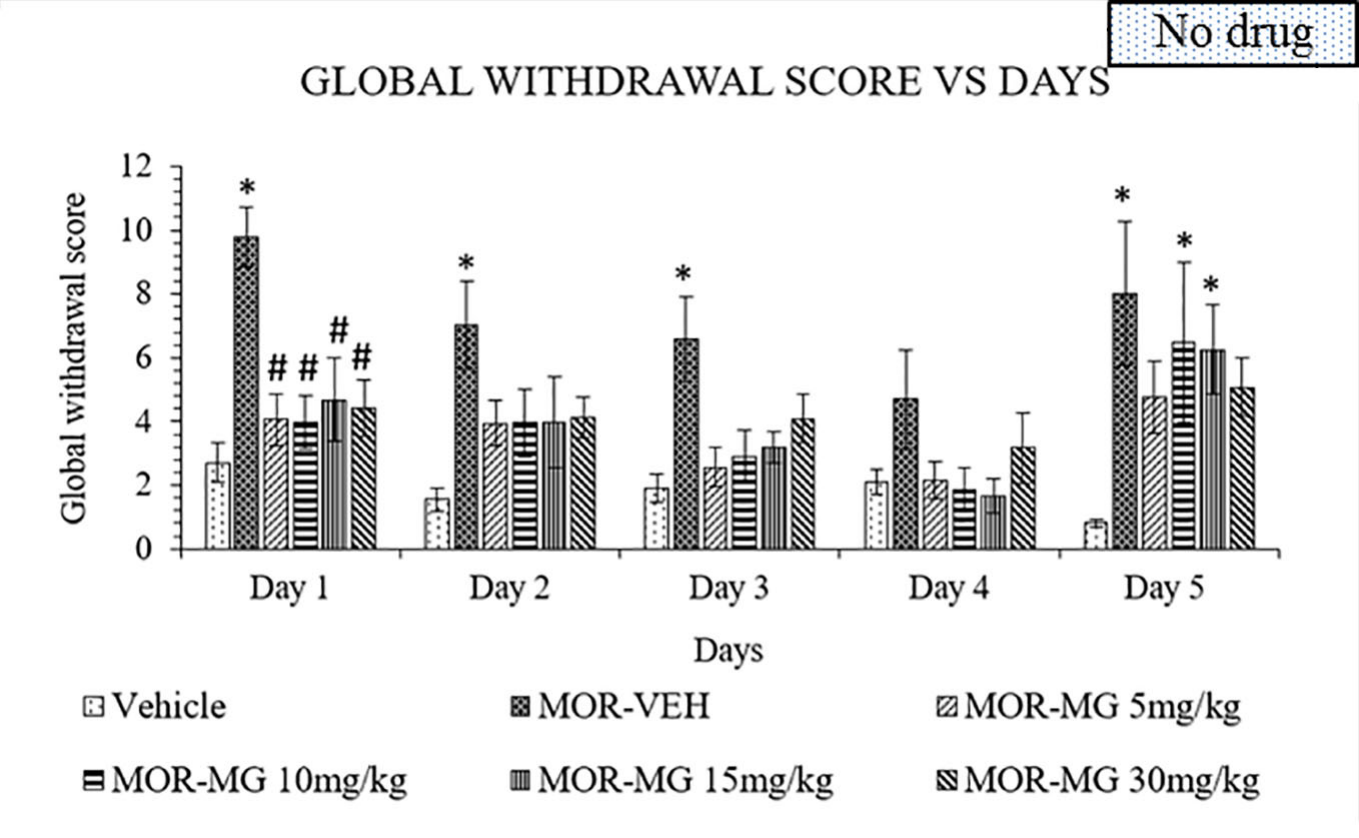
Mitragynine (MIT) reduces behavioral signs of morphine withdrawal in rats. Data represent means (± SEM) of global withdrawal signs (n = 6/group; *p < 0.05, vs. Vehicle, ^#^p < 0.05 vs. morphine-vehicle, MOR-VEH).

The established model was used to induce withdrawal effects that were clearly observed in the morphine group. Mitragynine attenuated morphine withdrawal effects significantly on day 1 to 4 (**Figure 2**). A two-way ANOVA showed a significant treatment (F_5,150_ = 13.02, p < 0.0001) and day effect (F_4,150_ = 5.742, p = 0.0003), but no significant interaction (F_20,150_ = 0.8071, p = 0.7021). Substitution treatment was not given on day 5, which resulted in the appearance of withdrawal signs in all previously mitragynine treated groups. On day 5, both 10 and 15 mg/kg doses of mitragynine treatment showed significant difference compared to vehicle group (p < 0.05 vs vehicle). This might suggest that both 10 and 15 mg/kg mitragynine have dose-dependent activity since the withdrawal sign effects increased slightly after treatment cessation on day 5. On the other hand, 5 and 30 mg/kg doses of mitragynine treatment showed no significance compared to both vehicle and morphine groups (p > 0.05). These findings suggest that mitragynine effectively reduced morphine withdrawal effects over 1 to 4 days of substitution. However, the number of substitution days for mitragynine should be prolonged since reduction in withdrawal scores could not be seen after cessation on day 5. Both 5 and 30 mg/kg doses were the best doses to be chosen in future, since they are able to reduce withdrawal and induce minimal withdrawal sign on day 5, during abrupt cessation.

### Methadone Attenuates Morphine-Withdrawal Behavior

**Figure 3:**
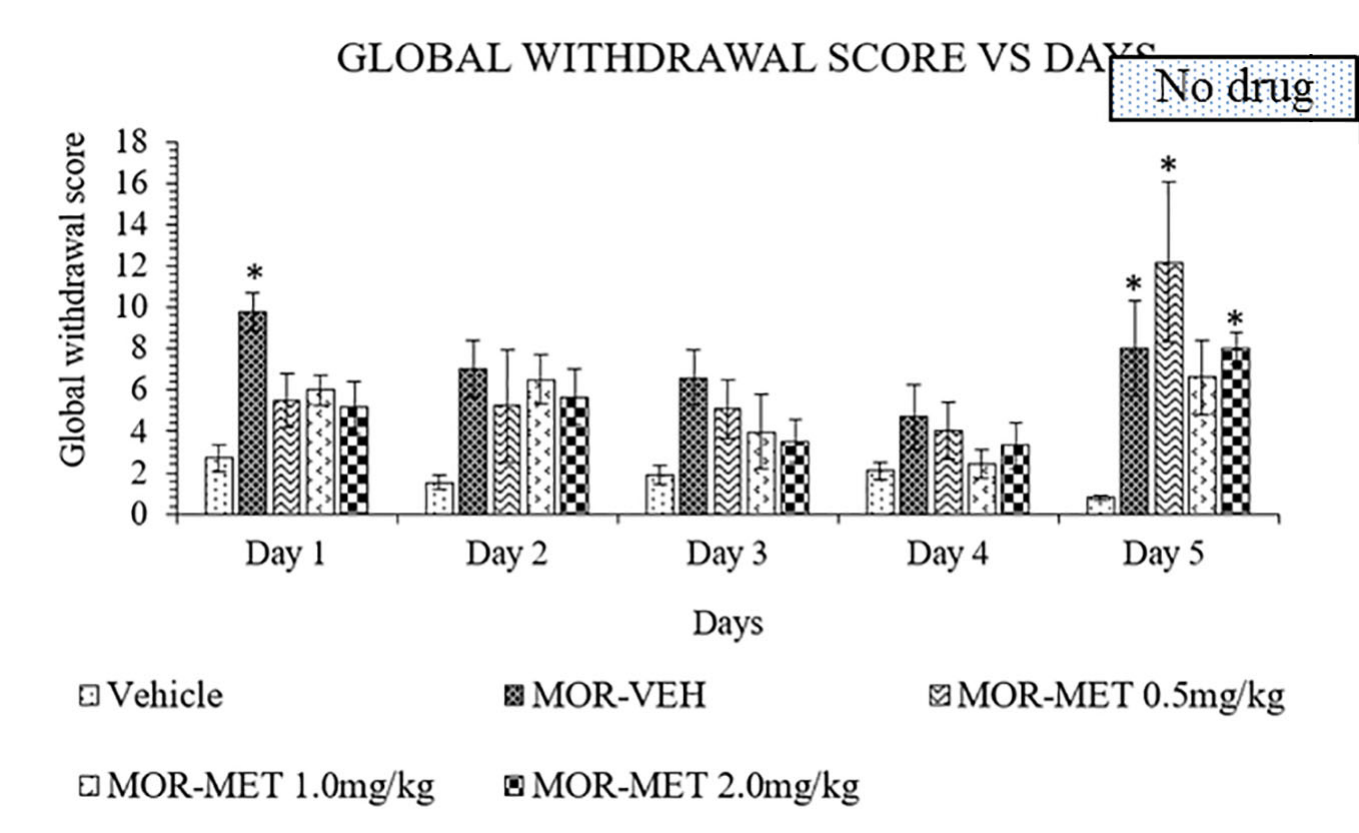
Methadone (MET) reduces behavioral signs of morphine withdrawal in rats. Data represent means (± SEM) of global withdrawal signs (n = 6/group; *p < 0.05, vs. Vehicle).

Methadone was also used as substitute treatment against morphine induced withdrawal rats for 4 days and abruptly stopped on day 5. A two-way ANOVA showed a significant treatment (F_4,__115_ = 8.756, p < 0.0001), and day effects (F_4,__115_ = 4.449, p = 0.0022), but no significant interaction between treatment groups and days (F_16,__115_ = 1.134, p = 0.3327). From day 1 to 4, no significant differences could be seen between methadone and vehicle groups for all methadone doses (**Figure 3**). Cessation of methadone treatment on day 5 yielded an increased in global withdrawal score for 0.5 and 2 mg/kg doses of methadone compared to vehicle groups. On the other hand, 1 mg/kg methadone showed a minimal increase of withdrawal sign. Thus, 4 days of substitution were insufficient to persistently reduce withdrawal signs in morphine withdrawn rats.

### Buprenorphine Attenuates Morphine-Withdrawal Behavior

**Figure 4:**
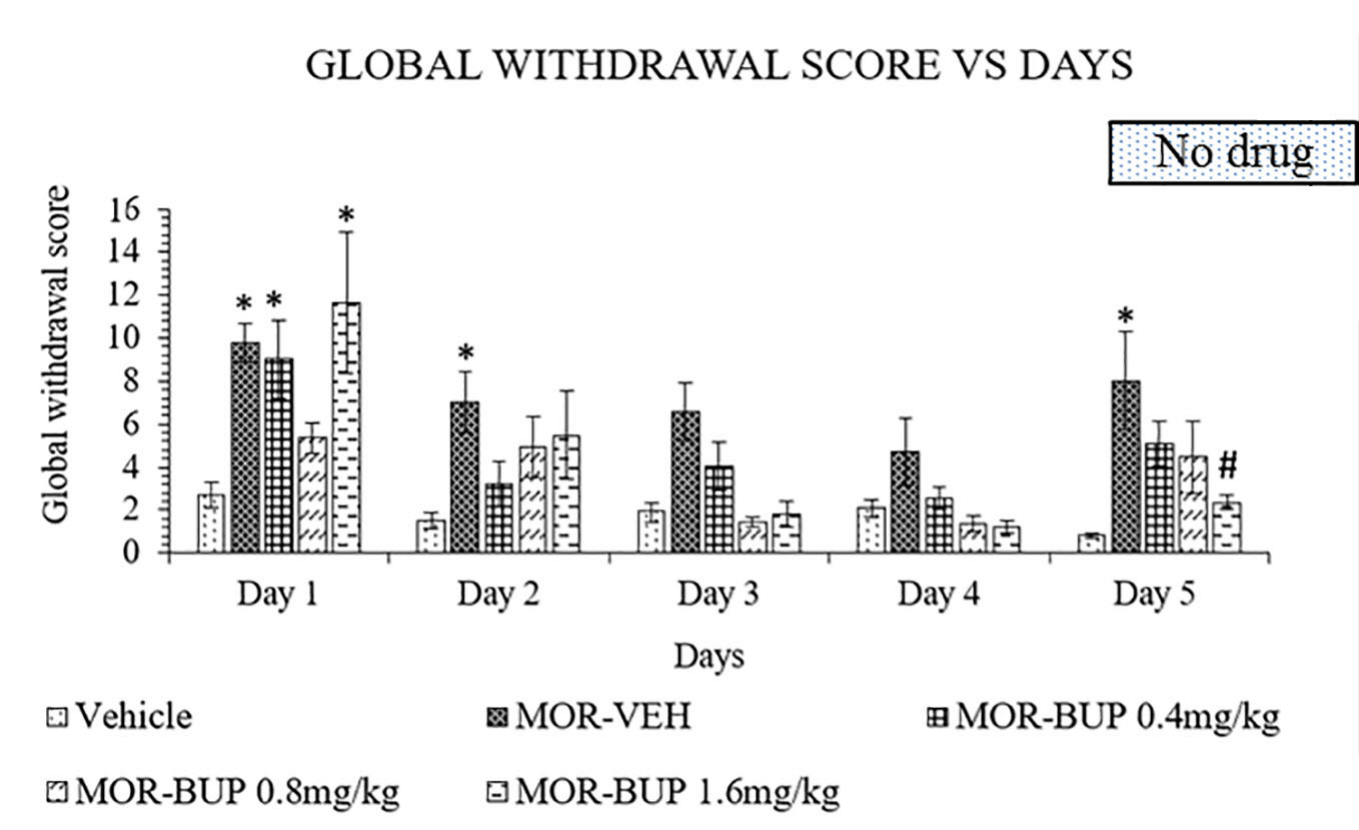
Buprenorphine (BUP) reduces behavioral signs of morphine withdrawal in rats. Data represent means (± SEM) of global withdrawal signs (n = 6/group; *p < 0.05, vs. Vehicle, ^#^p < 0.05 vs. morphine-vehicle, MOR-VEH).

Buprenorphine is another substitute treatment alternative for morphine withdrawal. Like in the previous experiments, treatment was continued for 4 days before abrupt cessation on day 5. A two-way ANOVA showed a significant treatment (F_4,110_ = 11.49, p < 0.0001), day effect (F_4,110_ = 12.22, p < 0.0001), and interaction between treatment and days (F_16,110_ = 1.803, p = 0.0393). Buprenorphine, however, failed to lower withdrawal score especially for doses 0.4 and 1.6 mg/kg on day 1 (**Figure 4**). The withdrawal scores were decrease on day 2 to 4 in both 0.4 and 1.6 mg/kg buprenorphine treatments compared to the vehicle group. While the 0.8 mg/kg dose of buprenorphine showed no significant effect on day 1 compared to the vehicle group, discontinuation of treatment on day 5 revealed no significant differences when compared to vehicle group. Although 1.6 mg/kg dose significantly reduce the withdrawal effect after cessation, buprenorphine still showed antagonistic effect on day 1.

### Hematological and Biochemical Analysis

Hematological and biochemical analysis of the blood samples were taken on day 5 and results are presented in **Supplementary Tables 4** and **5**. All results were within the normal reference range (60–63), indicating no effects of the treatments on blood parameters.

### Histopathology

All groups were further examined for the histopathological changes in the organs such as lung, heart, liver and kidney. Cross examination *via* the microscopic structures of the heart and lung showed no differences between the vehicle and treatment groups. However, histoarchitecture of the kidney revealed observable cellular damage in the MOR-VEH, MOR-MG 5 mg/kg, and MOR-MET 1 mg/kg treated groups as compared to VEH group. The kidney of control group (VEH) revealed normal glomerulus, Bowman's capsule and renal corpuscle. However, MOR-VEH, MOR-MG 5 mg/kg, and MOR-MET 1 mg/kg treated rats showed inflammation in the renal tubules, shrinkage of glomeruli with eroded Bowman's capsule with hemorrhage and increasing renal spaces were occasionally observed in the kidney, which was most clearly seen in the MOR-MG 5 mg/kg treated group. Cross examinations of MOR-MG 5 and 30 mg/kg, MOR-MET 1 mg/kg, MOR-BUP 0.8 mg/kg treated, and VEH groups preserved hepatic architecture with well-organized hepatic cell and central vein. Normal appearance of central vein and hepatic sinusoids lined by endothelial cells with normal radiating hepatocytes was observed in all groups. However, MOR-VEH treated rats showed slight degeneration in the liver with dark eosinophilic cytoplasm (**Supplementary Table 6**).

## Discussion

Opiate abuse is a major health problem in many countries. Managing opiate withdrawal appears as a key challenge in the treatment of opiate addicts. There are substitution drugs that can be used, but all come with major drawbacks and risks so that an improvement is much warranted. Here we report that the main psychoactive alkaloid of the kratom plant, mitragynine, mitigates morphine withdrawal symptoms. Although preclinical studies have shown that mitragynine may have an abuse potential and adverse cognitive effects by itself (23, 36, 43, 64), field studies have shown that these effects emerge usually at doses way higher than what humans voluntarily consume (65, 66). In addition, we did not find negative effects of mitragynine substitution on hematological, biochemical, and tissue level in this study, which confirms field studies that report mild impairments even after long term chronic use (67, 68). These findings may suggest considering further exploration of mitragynine as a potential substitution drug.

Various methods have been established to model morphine withdrawal in rats including implantation of morphine pellet (55, 56, 69–71), infusion method *via* implantation of intravenous catheters (72, 73), subcutaneous mini osmatic pumps (74, 75), drinking morphine solution containing water or sucrose (76–78), morphine-admixed food (79), or subcutaneous injection (80–83) and intraperitoneal route (54, 84, 85). However, all these methods have certain disadvantages. For instance, pellet and mini osmotic pump implantation require surgery. The implantation and removal of the pellets from animals' body may develop additional stress to the animals including the chances of getting infection at the implantation sites. Drinking morphine may develop a bitter taste and leads to considerable inter-individual differences in consumption between animals. Additionally, oral intake of morphine that achieves maximum plasma levels over a 4-h period with 83% bioavailability (86), can delay and lessen the effects compared to, e.g. parental administration. A substance's absorption, bioavailability and metabolism are affected by its chemical and physical properties as well as by its doses and route of administrations (87). In general, the extent of absorption reflects the total amount of drug entering the body which can be considered through this following order; intravenous > intraperitoneal > intramuscular> subcutaneous > oral (88). Therefore, the intraperitoneal route of administration is commonly used in small laboratory animals.

In the present study, the model used a slightly modified version of the Rahman et al. (54) model. The original model treated with morphine for 7 days. Further optimization, however, revealed that rats showed the full spectrum of withdrawal signs already after 6 days of morphine treatment. This would save time and cost. The maximum dose used in our model was 100 mg/kg morphine at day 6 compared to 180 mg/kg on day 6 (54). By reducing the number of days and dose, the mortality rate could be significantly reduced. The present model also evaluates the natural recovery from morphine withdrawal without any substitution treatment (**Figure 1**). Withdrawal signs include chewing, head shakes, exploring, teeth chattering, wet dog shakes, writhing, squeaking on touch, and hostility on handling. Each behavior develops its own pattern and duration of emergence. For example, in the present study, wet dog shakes behavior was more significant in the middle of the abstinence days. Other finding also reported that wet dog shakes act as a “recessive” behavior which declines when “dominant” signs, like teeth chattering, increase (55). Generally, the propensity of withdrawal signs was comparable to other studies (54, 55). Therefore, in order to better control the high variability of the individual signs of spontaneous abstinence, a global withdrawal score was calculated. The scores were multiplied by a weighing factor (**Supplementary Table 2**). When the global withdrawal scores were calculated, the clarification of the withdrawal signs during abstinence was more reliable (59), and was used in the substitution treatment part of the present study.

The present withdrawal model revealed that the most severe withdrawal signs was exhibited 24 h after the last morphine intake. The severity and duration of opioid withdrawal symptoms varies as a function of half-life of the opioid, the duration of opioid use, and patient-specific characteristics including health status. Abrupt cessation of short-acting opioids (e.g. heroin, hydrocodone, and oxycodone) is associated with severe opioid withdrawal signs that typically begin within 12 h after the last dose, peak at 36–72 h, and gradually taper off over the following 4–7 days (89). In rats, regardless of their route of administration, the plasma half-life of morphine is approximately 115 min (90). The entire dose administered was almost cleared from the body by four to five cycle of half-life (91). Thus, morphine would be mostly cleared from the body in approximately 8 to 10 h in rats. The withdrawal signs emerged after 12 h of the last morphine dose and reached their peak after 24 h thereafter. The study was continued after cessation until the rats were fully recovered from withdrawal signs. The effect of withdrawal gradually lessens from day 17 until day 28, where withdrawal effect fully disappears. The present study is in line with Gold et al. (71), in which withdrawal became increasingly intense up to 24 h post-implant and lasted up to 13 days post-implant, with almost no abstinence signs were observed after 18 days post-implant. Similar findings were also reported in a study by Goeldner et al. (92) which revealed that physical dependence to morphine is no longer exist after 4 weeks of abstinent period. In humans, opioid withdrawal signs will generally resolve after 5–14 days, depending on the half-life of the respective opioid (89). Most patients would not be able to cope with opioid withdrawal without proper substitution treatment. With regard to the above information, this model can induce acute withdrawal signs after 24 h and recover naturally after 16 days of abstinent without any treatment with drugs.

Kratom efficacy in managing withdrawal signs has been repeatedly reported in the literatures. Earliest reports by Burkill in 1935 had been widely acknowledged in kratom's research for the treatment of opium withdrawal (93). In the present study, the main indole alkaloid of kratom, mitragynine was capable to lessen withdrawal signs in morphine dependent rats. This finding was in line with a previous study which reported that mitragynine had low abuse liability and could attenuate the acquisition and expression of morphine-induced conditioned place preference (94). In addition, based on *in vitro* study stated that mitragynine acts at mu- and delta- opioid receptors (95). It can be readily assumed that analgesic effects as well as the mitigation of opiate withdrawal signs are mediated through these receptors (96). Moreover, antinociceptive activity of mitragynine has been proposed to be mediated by activation of descending noradrenergic and serotonergic pathways in the spinal cord (97). In another study, Khor et al. (98) suggested that mitragynine may attenuate stress-related swimming behaviors in morphine-withdrawn zebrafish. The mitigation of corticotropin releasing factor receptors and prodynorphin mRNA expression in zebrafish brain during the morphine withdrawal phase, indicates mitragynine's capability of reducing anxiety *via* the stress-related corticotropin pathway during opiate withdrawal. Thus, these findings might be related to the mechanism in which mitragynine mitigates withdrawal signs in morphine withdrawn rats.

Methadone has been used as an alternative substitution and maintenance therapy for the treatment of opioid addicts. Methadone acts as mu agonist, which accounts for its analgesic and antitussive properties with side effects like respiratory depression, decreased bowel motility, miotic pupils, nausea, and hypotension. In the present study, the methadone maintenance therapy at doses of 0.5, 1, and 2 mg/kg reduced withdrawal signs in morphine withdrawn rats. Abrupt cessation of methadone on day 5 revealed that rats given a 1.0 mg/kg methadone dose showed a minimal increase in withdrawal signs. On the other hand, rats given 0.5 and 2 mg/kg showed again an increase in withdrawal signs. A previous study by Ruiz et al. (49) suggested that 2 mg/kg of methadone was able to block opiate dependence following naloxone administration after methadone substitutive treatment. This might be due to differences in the animal model used. The half-life of methadone in rats is 1.5 ± 0.4 h (47) and 24-36 h in human after oral intake (99). Elimination of methadone is much more rapid in rats compared to humans (100, 101). Eight hours after last methadone dose, withdrawal signs could be seen, similar to the morphine group without treatment. Hence, a longer period of methadone maintenance therapy may possibly suppress the withdrawal signs in morphine withdrawn rats.

Buprenorphine substitution treatment 24 h after the last morphine dose indicates the failure of both doses 0.4 and 1.6 mg/kg buprenorphine in suppressing withdrawal signs on day 1 compared to the vehicle group. However, 0.8 mg/kg buprenorphine treatment showed no significant difference when compared to vehicle group, suggesting that this dose was able to suppress the morphine withdrawal effects on day 1. A previous study reported that the antinociceptive effect of buprenorphine reaches its peak at an approximate dose of 0.5 mg/kg s.c. (102). The 1.6 mg/kg buprenorphine dose on day 1 surprisingly caused a small increase in withdrawal score in morphine withdrawn rats. This might be due to the antagonistic effect of buprenorphine. Buprenorphine is a partial opioid agonist that can act as an antagonist under certain condition due to its low intrinsic activity (103). A study from Dum and Herz (102) showed that buprenorphine was able to precipitate withdrawal in rats at doses above 1 mg/kg and 30 mg/kg was the greatest withdrawal-precipitating potency. Compared to full agonist drug such as methadone, buprenorphine has a lower ceiling effect to euphoria (104). It might also be used as a safer intervention, especially for its ceiling effect on respiratory depression (105). Cessation of buprenorphine maintenance therapy on day 5 increases withdrawal signs except for the previously 1.6 mg/kg buprenorphine treated group. A study on buprenorphine pharmacokinetics in rats suggested that its plasma half-life is 5.3 h after bolus intravenous administration (106). Since the final scoring was performed 12 h after the last dose, the remaining buprenorphine in the plasma could still attenuate withdrawal signs, which might explain the minimal increase in withdrawal signs in comparison to methadone and mitragynine on day 5.

Morphine-induced damages on the vital organs (liver, kidney, heart, and lung) are frequently reported in rodent models, especially at high dose for a short period (for example, higher than 120 mg/kg/day) or at lower dose for chronic use (107). In this study, histopathological and biochemical changes due to the usage of morphine and/or the substitution drugs in selected vital organs were assessed. In all groups, particularly the morphine group, no histopathological changes were observed, indicating that the selected doses of drugs administration at determined duration, were too small to cause histopathological damage but sufficient to show signs of drug intoxication. This study also further investigated the effects of morphine and drug substitutes administration on hematological and biochemical indices in rats. Similarly, no apparent changes observed in all parameters. This data further confirmed the selected doses of morphine is effective in leading to significant phenotypic readout (upon morphine withdrawal) with low-to-no toxicity effect. Behavioral outcomes are associated with neural circuits, and, hence, morphine-induced neurochemical changes is warrant for further investigations.

Although all the results for hematological and biochemical analysis of the blood samples were within the normal reference range values, significant effects of treatment against the PLT and alkaline phosphatase were observed in this study (**Supplementary Tables 4** and **5**). The significant effects of treatment against the PLT could be considerably affected in healthy rats by biological variations such as variability between individual rats and temporal variation (108) after the treatment which warrants further investigations. In addition, MOR-MG 5, 15 and 30 mg/kg, MOR-BUP 0.8 mg/kg, and MOR-MET 0.5 mg/kg treatments were significantly affected the serum liver enzyme activity of alkaline phosphatase in this study. Serum liver enzyme activity was used to monitor the presence or absence of liver injury after the rats were subjected to various treatments in this study. Furthermore, the findings of this study also suggest that MOR-MG 5, 15, and 30 mg/kg, MOR-BUP 0.8 mg/kg and MOR-MET 0.5 mg/kg treatments might mildly affect the structural integrity of hepatocellular membrane of healthy rats, thus facilitate the alkaline phosphatase leakage into the blood circulation (109). Moreover, the increased in the activities of alkaline phosphatase observed in this study might correspond to mild ignorable liver damage induced in the treated rats since the increment still within the normal reference range values. However, further, prolong detailed studies are warranted to directly examine the effects of treatments against alkaline phosphatase leakage.

Based on the above findings, it is suggested that long period of maintenance therapy is required for the treatment of morphine dependent rats. Mitragynine might be a possible new alternative therapy for opioid addicts in replacement of methadone and buprenorphine. Since mitragynine is extracted from kratom leaves, it can be accessed easily and cheaper as compared to other drugs. Methadone replacement therapy is still subjected to overdose and fatalities due to a lack of ceiling effects at the level of respiratory depression and sedation, as seen in morphine overdose (110). The methadone dose varies between patients and it is very hard to identify the best dose to be given to patients. On the other hand, buprenorphine is potentially safer than methadone. As a partial agonist, it produces less physical dependence compared to methadone. Yet, buprenorphine develops poor retention and is less satisfying for patients since they develop a mild withdrawal syndrome when displacing heroin. An overdose of an opioid can cause respiratory depression and death. These side effects have been linked to the alteration of MOR regulation. Previous studies have shown that mice lacking the G-protein-coupled receptor regulatory protein, β-arrestin 2, display profoundly altered morphine responses. β-arrestin 2 knockout mice enhanced and prolonged morphine analgesia, attenuated the respiratory suppression and acute constipation (32). In *in vitro* study conducted by Kruegel et al. (29), mitragynine acts as a partial agonist at human MORs and did not result in recruitment of β-arrestin 2 providing a potential mechanism for low abuse and respiratory depression liability. The effect of mitragynine might be mediated through opioid receptor or interaction with other opioid agonist. Several studies proposed the possibility that mitragynine effects were mediated through opioid receptors (29, 41, 111, 112), Hiranita et al. (113) however suggested that mitragynine has different pharmacological mechanism as compared to morphine. Its antinociceptive effect is not mediated *via* opioid receptor since naltrexone which antagonize morphine's effects on schedule-controlled responding and thermal response latency did not alter mitragynine's effects significantly (113). Furthermore, a study by Hemby et al. (114) showed that mitragynine is a good candidate for pharmacotherapies, since it reduces morphine intake.

In conclusion, the morphine withdrawal model induced withdrawal signs for 16 days in rats. Four-day replacement treatment with mitragynine attenuated the withdrawal symptoms significantly, suggesting that mitragynine is able to reduce morphine withdrawal symptoms similar to methadone and burprenorphine. These findings suggest that mitragynine, the major indole alkaloid of kratom, may be used as a potential treatment of opioid addiction-related withdrawal. Further research is required including clinical trials to evaluate the application of mitragynine as a supplementary treatment of opioid addiction in humans.

## Implications

The present study suggests that mitragynine may serve as an alternative treatment for opiate withdrawal effects as they occur in opiate addiction. Although mitragynine may possess some addictive properties on its own, it may, in low-medium doses, in which humans voluntarily use it, help to manage opiate addiction. The current report details the efficacy in comparison to methadone and buprenorphine. While mitragynine is equally effective in reducing opiate withdrawal effects in rats, it may be the safer drug with less undesired side-effects.

## Limitation and Future Research

A general limitation of this study is the experimenter administered substitution treatment, which does not allow to make predictions about acceptance of a mitragynine pharmacotherapy by human opioid addicts (115). While a morphine withdrawal state was induced in this study, it is still not the full picture of an opioid addiction, as manifested in the current opioid crisis. Whether mitragynine is equally effective in addicted individuals still has to be evaluated. The current study may also encourage research in the question of how mitragynine affects other addiction-related behaviors, such as drug seeking and self-administration.

## Data Availability Statement

All datasets generated for this study are included in the article/**Supplementary Material**.

## Ethics Statement

The animal study was reviewed and approved by Animal Ethics Committee of Universiti Sains Malaysia.

## Author Contributions

ZH, SM, and CM planned the study. RH, CP, and SS performed the experiments and analyzed the data together with ZH. RH, CM, and ZH wrote the first draft of the manuscript. All the authors commented on the manuscript. All the authors contributed to and have approved the final manuscript.

## Conflict of Interest

The authors declare that the research was conducted in the absence of any commercial or financial relationships that could be construed as a potential conflict of interest.
