# Supplementary material for: Mitragynine Attenuates Morphine Withdrawal Effects in Rats—A Comparison With Methadone and Buprenorphine
Source: Front Psychiatry. 2020 May 7;11:411. doi: 10.3389/fpsyt.2020.00411 (PMC7221179; doi:10.3389/fpsyt.2020.00411)
Supplement: Supplementary file 1 [file DataSheet_1.pdf]

Supplementary Table 1 : Dosing schedule to induce morphine-dependence related withdrawal signs in rats.

Days	Time (h)	Dose (mg/kg; i.p.)
**1st**	4.00 pm	10
**2nd**	9.00 am	10
	4.00 pm	10
**3rd**	9.00 am	20
	4.00 pm	20
**4th**	9.00 am	30
	4.00 pm	30
**5th**	9.00 am	40
	4.00 pm	40
**6th**	9.00 am	50
	4.00 pm	50
**7th**	Abruptly stop to see withdrawal symptoms	1st scored after 24 hours

Supplementary Table 2 : The counted signs and checked signs with the respective weighing factors for the evaluation of morphine-withdrawal severity in rats.

Counted signs	Weighing factors	Checked signs (Checked every 10 minutes)	Weighing factors
Chewing	2	Squeaking on touch	1
Head shakes	2	Hostility on handling	1
Exploring	1	Diarrhoea	1
Digging	2		
Yawning	2		
Teeth chattering	2		
Wet dog shakes	2		
Writhing	2		

Morphine withdrawal model

Individual behaviour scores after spontaneous morphine withdrawal

The individual signs which contributed to the overall withdrawal scores are shown in Suppl. Tab 3. Counted signs consist of chewing, head shake, exploring, digging, yawning, teeth chattering, wet dog shake and writhing. On the other hand, squeaking on touch, hostility on handling and diarrhoea were checked and counted at 10 minutes interval. In our set up, there were occasional episodes of chewing, exploring, digging and yawning in the vehicle group. For morphine-withdrawn rats, two-way ANOVA revealed a significant treatment effect for counted signs; chewing ($F_{(1, 168)} = 58.62$, $P < 0.0001$), head shakes ($F_{(1, 168)} = 279.3$, $P < 0.0001$), exploring ($F_{(1, 168)} = 176.9$, $P < 0.0001$), digging ($F_{(1, 168)} = 9.806$, $P = 0.0021$), yawning ($F_{(1, 168)} = 3.990$, $P = 0.0474$), teeth chattering ($F_{(1, 168)} = 622.1$, $P < 0.0001$), wet dog shake ($F_{(1, 168)} = 97.61$, $P < 0.0001$), writhing ($F_{(1, 168)} = 32.92$, $P < 0.0001$ whereas for checked signs; squeaking on touch ($F_{(1, 168)} = 74.58$, $P < 0.0001$), hostility on handling ($F_{(1, 168)} = 56.61$, $P < 0.0001$) and diarrhoea ($F_{(1, 168)} = 21.41$, $P < 0.0001$). For day factor, significant effect was observed only in the case of chewing ($F_{(27, 168)} = 2.976$, $P < 0.0001$), head shakes ($F_{(27, 168)} = 13.92$, $P < 0.0001$), exploring ($F_{(27, 168)} = 2.152$, $P = 0.0018$), teeth chattering ($F_{(27, 168)} = 20.25$, $P < 0.0001$), wet dog shakes ($F_{(27, 168)} = 1.911$, $P = 0.0073$), writhing ($F_{(27, 168)} = 2.079$, $P = 0.0027$), hostility on handling ($F_{(27, 168)} = 2.861$, $P < 0.0001$) and diarrhoea ($F_{(27, 168)} = 1.718$, $P = 0.0211$), but not in digging, yawning and squeaking on touch behaviours ($p>0.05$). Significant interaction between treatment and days were seen for behaviours such as chewing ($F_{(27, 168)} = 2.376$, $P = 0.0005$), head shakes ($F_{(27, 168)} = 13.92$, $P < 0.0001$), exploring ($F_{(27, 168)} = 1.840$, $P = 0.0108$), teeth chattering ($F_{(27, 168)} = 20.25$, $P < 0.0001$), wet dog shakes ($F_{(27, 168)} = 1.911$, $P = 0.0073$), writhing ($F_{(27, 168)} = 2.079$, $P = 0.0027$), hostility on handling ($F_{(27, 168)} = 2.861$, $P < 0.0001$) and diarrhoea ($F_{(27, 168)} = 1.718$, $P = 0.0211$) (Suppl. Tab 3). No changes in body weight were observed throughout the studies (data not shown).

Supplementary Table 3: Individual signs of spontaneous morphine withdrawal (chewing, head shakes, exploring, digging, yawning, teeth chattering, wet dog shakes, writhing, squeaking on touch, hostility on handling, diarrhoea) during 28 abstinence days, in vehicle and morphine (MOR) treated rats. Data represent means (± SEM) of individual behavioural signs (n=6/group; *p<0.05, **p<0.01, ***p<0.001 vs. Vehicle) within 28 days.

Signs of withdrawal syndrome							
**Days**	**Group**	**Chewing**	**Head shakes**	**Exploring**	**Digging**	**Yawning**	**Teeth chattering**
Day 1	Vehicle	5 ± 1.3	0 ± 0	17.25 ± 1.9	0 ± 0	0 ± 0	0 ± 0
	MOR	23 ± 6.6*	18.5 ± 1.26***	46.3 ± 8.4*	2.5 ± 1.5	0 ± 0	5.5 ± 0.5***
Day 2	Vehicle	2 ± 0.8	0 ± 0	15.25 ± 3.1	0 ± 0	0 ± 0	0 ± 0
	MOR	26.5 ± 8.5***	17.5 ± 1.7***	24.75 ± 11.6	2.5 ± 2.5	0 ± 0	4.5 ± 0.5***
Day 3	Vehicle	4 ± 0.8	0 ± 0	2.75 ± 0.9	0 ± 0	0 ± 0	0 ± 0
	MOR	31 ± 15.8***	12.5 ± 1.9***	27.25 ± 9.0	1 ± 1.0	0 ± 0	4 ± 0.8***
Day 4	Vehicle	0 ± 0	0 ± 0	5.75 ± 0.6	0 ± 0	0 ± 0	0 ± 0
	MOR	7.5 ± 4.3	8.5 ± 1***	15 ± 3.8	0.5 ± 0.5	0.5 ± 0.5	3.5 ± 0.5***
Day 5	Vehicle	2 ± 0.8	0 ± 0	6.75 ± 1.3	0 ± 0	0 ± 0	0 ± 0
	MOR	17.5 ± 7.8	7.5 ± 1***	24 ± 5.7	0.5 ± 0.5	0.5 ± 0.5	11 ± 1.3***
Day 6	Vehicle	0.5 ± 0.5	0 ± 0	7.5 ± 1.2	0 ± 0	0 ± 0	0 ± 0
	MOR	11.5 ± 3.3	6 ± 1.4***	29.5 ± 6.8	1 ± 0.6	0.5 ± 0.5	7 ± 0.6***
Day 7	Vehicle	2 ± 0.82	0 ± 0	6.75 ± 1.11	0 ± 0	0 ± 0	0 ± 0
	MOR	7 ± 4.7	4.5 ± 1.5*	28.5 ± 7.0	0 ± 0	0.5 ± 0.5	7.5 ± 1***
Day 8	Vehicle	1 ± 1	0 ± 0	11.5 ± 4.3	0 ± 0	0 ± 0	0 ± 0
	MOR	14 ± 6.2	4 ± 1.4	33 ± 5.5	3.5 ± 3.5	0 ± 0	3 ± 1.3**
Day 9	Vehicle	2 ± 0.82	0 ± 0	10 ± 1.1	0 ± 0	0 ± 0	0 ± 0
	MOR	18.5 ± 8.5	8.5 ± 1.9***	52.25 ± 17.5***	0.5 ± 0.5	0 ± 0	5 ± 1***
Day 10	Vehicle	3 ± 0.58	0 ± 0	11.75 ± 0.3	2 ± 0.8	1.5 ± 0.5	0 ± 0
	MOR	6 ± 2.6	2.5 ± 0.5	37.5 ± 10.9	1 ± 1	1.5 ± 1.0	3 ± 0.6**
Day 11	Vehicle	3.5 ± 0.96	0 ± 0	6.5 ± 1.0	0.5 ± 0.5	0.5 ± 0.5	0 ± 0
	MOR	16 ± 8.5	4.5 ± 2.2*	52.5 ± 17.0***	1 ± 1	2.5 ± 2.5	5.5 ± 1***
Day 12	Vehicle	0 ± 0	0 ± 0	5.25 ± 0.6	0 ± 0	0 ± 0	0 ± 0
	MOR	13.5 ± 6.2	4.5 ± 2.2*	48.5 ± 15.8***	1 ± 1	2.5 ± 2.5	5 ± 1***
Day 13	Vehicle	2.5 ± 0.5	0 ± 0	8.5 ± 0.6	0 ± 0	1.0 ± 0.6	0 ± 0
	MOR	23.5 ± 8.5**	3.5 ± 3.5	35 ± 6.3	2 ± 1.4	1.0 ± 0.6	9 ± 0.6***

Days	Group	Chewing	Head shakes	Exploring	Digging	Yawning	Teeth chattering
Day 14	Vehicle	1.5 ± 0.96	0 ± 0	9.25 ± 0.5	0 ± 0	0.5 ± 0.5	0 ± 0
	MOR	11 ± 4.4	3.5 ± 1	45 ± 13.1**	0.5 ± 0.5	0 ± 0	10 ± 1.4***
Day 15	Vehicle	0.5 ± 0.5	0 ± 0	6.75 ± 1.2	1.0 ± 0.6	1.5 ± 0.5	0 ± 0
	MOR	16.5 ± 2.6	4.5 ± 1*	22.25 ± 0.5	1 ± 1	0.5 ± 0.5	1 ± 0.6
Day 16	Vehicle	0.5 ± 0.5	0 ± 0	7.25 ± 0.9	0.5 ± 0.5	0.5 ± 0.5	0 ± 0
	MOR	9.5 ± 5.5	3 ± 1.3	28.25 ± 5.2	3.5 ± 1.7	0 ± 0	10 ± 1.2***
Day 17	Vehicle	4 ± 0.82	0 ± 0	10.5 ± 1.2	1.5 ± 1.0	0.5 ± 0.5	0 ± 0
	MOR	3.5 ± 1.3	2 ± 1.4	21.5 ± 2.1	0 ± 0	1.5 ± 1.0	4.5 ± 1.5***
Day 18	Vehicle	2 ± 0.82	0 ± 0	8.25 ± 1.0	0.5 ± 0.5	1.5 ± 0.5	0 ± 0
	MOR	2.5 ± 1.3	1.5 ± 1	24.25 ± 7.3	0.5 ± 0.5	0 ± 0	1 ± 0.6
Day 19	Vehicle	0 ± 0	0 ± 0	4.25 ± 0.9	0 ± 0	0 ± 0	0 ± 0
	MOR	3 ± 1.3	1 ± 1	29 ± 5.2	0 ± 0	4 ± 2.4	3.5 ± 1***
Day 20	Vehicle	6 ± 1.41	0 ± 0	5.25 ± 0.5	0 ± 0	0 ± 0	0 ± 0
	MOR	1.5 ± 1	1 ± 1	32 ± 5.7	1.5 ± 1.5	0 ± 0	0 ± 0
Day 21	Vehicle	1.5 ± 1.5	0 ± 0	13.25 ± 2.5	0.5 ± 0.5	0.5 ± 0.5	0 ± 0
	MOR	1.5 ± 1	0 ± 0	36.25 ± 9.0	1.5 ± 1.5	0 ± 0	0 ± 0
Day 22	Vehicle	0.25 ± 0.3	0 ± 0	8.5 ± 2.5	0 ± 0	0.5 ± 0.5	0 ± 0
	MOR	1 ± 0.6	0 ± 0	30.75 ± 5.5*	1.5 ± 1.5	0 ± 0	0 ± 0
Day 23	Vehicle	0 ± 0	0 ± 0	6 ± 1.2	0 ± 0	0 ± 0	0 ± 0
	MOR	1.5 ± 1	0 ± 0	29.25 ± 4.0	1.5 ± 1.5	0.5 ± 0.5	0 ± 0
Day 24	Vehicle	1.5 ± 1	0 ± 0	4.75 ± 0.6	1.5 ± 0.5	1 ± 0.6	0 ± 0
	MOR	1.5 ± 1	0 ± 0	31.25 ± 6.3*	1.5 ± 1.5	0 ± 0	0 ± 0
Day 25	Vehicle	1.5 ± 1	0 ± 0	8.5 ± 2.3	0 ± 0	0 ± 0	0 ± 0
	MOR	2.5 ± 1.5	0 ± 0	22 ± 5.4	0 ± 0	1.5 ± 1.0	0 ± 0
Day 26	Vehicle	0 ± 0	0 ± 0	3.75 ± 0.6	0 ± 0	0 ± 0	0 ± 0
	MOR	2.5 ± 1.5	0 ± 0	11.75 ± 2.9	0.5 ± 0.5	3 ± 3	0 ± 0
Day 27	Vehicle	1.5 ± 0.5	0 ± 0	7.75 ± 0.5	0.5 ± 0.5	0 ± 0	0 ± 0
	MOR	0 ± 0	0 ± 0	7.75 ± 2.8	0 ± 0	1 ± 1	0 ± 0
Day 28	Vehicle	0 ± 0	0 ± 0	15.25 ± 0.85	0 ± 0	0 ± 0	0 ± 0
	MOR	0 ± 0	0 ± 0	13.25 ± 1.7	0 ± 0	0 ± 0	0 ± 0

Signs of withdrawal syndrome						
**Days**	**Group**	**Wet dog Shakes**	**Writhing**	**Squeaking on touch**	**Hostility on handling**	**Diarrhoea**
Day 1	Vehicle	0 ± 0	0 ± 0	0 ± 0	0 ± 0	0 ± 0
	MOR	19 ± 8.6	7 ± 3.5***	3 ± 0**	3 ± 0***	0 ± 0
Day 2	Vehicle	0 ± 0	0 ± 0	0 ± 0	0 ± 0	0 ± 0
	MOR	11.5 ± 2.9	3.5 ± 2.9	2.25 ± 0.8	2 ± 0.7*	3 ± 0***
Day 3	Vehicle	0 ± 0	0 ± 0	0 ± 0	0 ± 0	0 ± 0
	MOR	14 ± 2.4	2.5 ± 1.5	2 ± 0.7	2.25 ± 0.8**	0 ± 0
Day 4	Vehicle	0 ± 0	0 ± 0	0 ± 0	0 ± 0	0 ± 0
	MOR	8 ± 3.6	4.5 ± 2.9*	1.5 ± 0.9	0 ± 0	0 ± 0
Day 5	Vehicle	0 ± 0	0 ± 0	0 ± 0	0 ± 0	0 ± 0
	MOR	18 ± 10.4	4 ± 1.4	1.75 ± 0.8	1.5 ± 0.9	0.75 ± 0.8
Day 6	Vehicle	0 ± 0	0 ± 0	0 ± 0	0 ± 0	0 ± 0
	MOR	14.5 ± 3.4	0.5 ± 0.5	0.75 ± 0.8	1.5 ± 0.9	0.25 ± 0.3
Day 7	Vehicle	0 ± 0	0 ± 0	0 ± 0	0 ± 0	0 ± 0
	MOR	7 ± 0.8	1 ± 0.6	2.25 ± 0.8	1.5 ± 0.9	0.75 ± 0.8
Day 8	Vehicle	0 ± 0	0 ± 0	0 ± 0	0 ± 0	0 ± 0
	MOR	11.5 ± 3.3	3.5 ± 1.7	1.5 ± 0.9	1.5 ± 0.9	0.5 ± 0.5
Day 9	Vehicle	0 ± 0	0 ± 0	0 ± 0	0 ± 0	0 ± 0
	MOR	17.5 ± 2.2	4 ± 2.4	2.25 ± 0.8	2.25 ± 0.8**	0.75 ± 0.8
Day 10	Vehicle	0 ± 0	0 ± 0	0 ± 0	0 ± 0	0 ± 0
	MOR	10 ± 1.8	1.5 ± 0.5	2.5 ± 0.5*	2.25 ± 0.8**	0.75 ± 0.8
Day 11	Vehicle	0 ± 0	0 ± 0	0 ± 0	0 ± 0	0 ± 0
	MOR	39.5 ± 21.7***	1 ± 0.6	0.75 ± 0.8	0.75 ± 0.8	0 ± 0
Day 12	Vehicle	0 ± 0	0 ± 0	0 ± 0	0 ± 0	0 ± 0
	MOR	33.5 ± 15.8***	1 ± 0.6	0.75 ± 0.8	0.75 ± 0.8	0 ± 0
Day 13	Vehicle	0 ± 0	0 ± 0	0 ± 0	0 ± 0	0 ± 0
	MOR	31 ± 15.0***	0.5 ± 0.5	1.5 ± 0.9	1 ± 0.7	0 ± 0

Signs of withdrawal syndrome						
**Days**	**Group**	**Wet dog Shakes**	**Writhing**	**Squeaking on touch**	**Hostility on handling**	**Diarrhoea**
Day 14	Vehicle	0 ± 0	0 ± 0	0 ± 0	0 ± 0	0 ± 0
	MOR	21 ± 8.2	0 ± 0	1.5 ± 0.9	1 ± 0.7	0 ± 0
Day 15	Vehicle	0 ± 0	0 ± 0	0 ± 0	0 ± 0	0 ± 0
	MOR	20.5 ± 5.4*	0.5 ± 0.5	0.75 ± 0.8	0 ± 0	0 ± 0
Day 16	Vehicle	0 ± 0	0 ± 0	0 ± 0	0 ± 0	0 ± 0
	MOR	7 ± 1.7	0.5 ± 0.5	0.75 ± 0.8	0 ± 0	0.5 ± 0.5
Day 17	Vehicle	0 ± 0	0 ± 0	0 ± 0	0 ± 0	0 ± 0
	MOR	15 ± 3.3	0.5 ± 0.5	0.75 ± 0.8	0 ± 0	0.25 ± 0.3
Day 18	Vehicle	0 ± 0	0 ± 0	0 ± 0	0 ± 0	0 ± 0
	MOR	9 ± 2.4	0 ± 0	0.75 ± 0.8	0 ± 0	0.75 ± 0.8
Day 19	Vehicle	0 ± 0	0 ± 0	0 ± 0	0 ± 0	0 ± 0
	MOR	22.5 ± 7.2	3.5 ± 1.7	2.25 ± 0.8	0.75 ± 0.8	0 ± 0
Day 20	Vehicle	0 ± 0	0 ± 0	0 ± 0	0 ± 0	0 ± 0
	MOR	5.5 ± 2.9	0 ± 0	0.75 ± 0.8	0 ± 0	0.75 ± 0.8
Day 21	Vehicle	0 ± 0	0 ± 0	0 ± 0	0 ± 0	0 ± 0
	MOR	4.5 ± 3.2	0 ± 0	0.75 ± 0.8	0 ± 0	0.5 ± 0.5
Day 22	Vehicle	0 ± 0	0 ± 0	0 ± 0	0 ± 0	0 ± 0
	MOR	5.5 ± 2.9	0 ± 0	0.75 ± 0.8	0 ± 0	0 ± 0
Day 23	Vehicle	0 ± 0	0 ± 0	0 ± 0	0 ± 0	0 ± 0
	MOR	7 ± 2.6	0 ± 0	0.75 ± 0.8	0 ± 0	0.5 ± 0.5
Day 24	Vehicle	0 ± 0	0 ± 0	0 ± 0	0 ± 0	0 ± 0
	MOR	5.5 ± 2.9	0 ± 0	0.75 ± 0.8	0 ± 0	0.5 ± 0.5
Day 25	Vehicle	0 ± 0	0 ± 0	0 ± 0	0 ± 0	0 ± 0
	MOR	6 ± 1.6	0 ± 0	0.75 ± 0.8	0 ± 0	0.75 ± 0.8
Day 26	Vehicle	0 ± 0	0 ± 0	0 ± 0	0 ± 0	0 ± 0
	MOR	3.5 ± 0.5	0 ± 0	0 ± 0	0 ± 0	0 ± 0
Day 27	Vehicle	0 ± 0	0 ± 0	0 ± 0	0 ± 0	0 ± 0
	MOR	2 ± 0	0 ± 0	0 ± 0	0 ± 0	0 ± 0
Day 28	Vehicle	0 ± 0	0 ± 0	0 ± 0	0 ± 0	0 ± 0
	MOR	0 ± 0	0 ± 0	0 ± 0	0 ± 0	0 ± 0

Supplementary Table 4: Haematological analysis on day 5 of all groups of treatment. Means ± SEM of n= 6 rats/group. *P<0.05, **P<0.01, ***P<0.001 vs. Vehicle, #P<0.05, ##P<0.01, ###P<0.001 vs. MOR analysed by two-way repeated measures ANOVA and Bonferroni post-hoc test.

**Groups**	**VEH**	**MOR-VEH**	**MOR-MG 5mg/kg**	**MOR-MG 10mg/kg**	**MOR-MG 15mg/kg**	**MOR-MG 30mg/kg**	**References value**
Total RBC (x 10^12/L)	7.025 ± 0.18	7.125 ± 0.2	7.38 ± 0.61	6.65 ± 0.12	6.15 ± 0.78	6.7 ± 0.23	6.39-8.01
Haemoglobin (gm/L)	158.25 ± 6.34	137.25 ± 3.57	131.75 ± 8.04	142.75 ± 5.54	140.25 ± 2.95	130.25 ± 6.16	135-159
PCV (%)	42 ± 1	41 ± 3	44 ± 3	39 ± 1	38 ± 1	39 ± 2	42-49
MCV (fL)	60.25 ± 1.03	56.75 ± 2.39	59.25 ± 0.48	58.75 ± 1.65	55.5 ± 1.55	57.75 ± 1.6	58.01-67.00
MCH (pg)	21 ± 0.41	19.5 ± 0.87	18 ± 0.41	21.5 ± 0.87	23.25 ± 2.98	19.5 ± 0.65	18.70-21.20
MCHC (g/L)	315 ± 6.45	345 ± 18.93	302.5 ± 4.79	367.5 ± 19.31	372.5 ± 17.5	342.5 ± 22.13	310-336
RDW (%)	15.83 ± 0.13	15.63 ± 0.38	14.03 ± 0.48	14.63 ± 0.11	15.9 ± 0.09	15.15 ± 0.38	13.03-16.57
Total WBC (x 10^9/L)	5.85 ± 1.59	6.2 ± 1.6	3.95 ± 1.42	4.65 ± 0.77	12.65 ± 5.64	7.13 ± 2.32	3.00-9.22
Lymphocytes (%)	57.25 ± 5.15	63.5 ± 4.29	79.25 ± 1.49	54.5 ± 7.35	61.25 ± 11.52	46.25 ± 10.69	51.8-89.7
Monocytes (%)	1.5 ± 0.65	5.75 ± 1.55	1.25 ± 0.25	4.75 ± 1.11	5.25 ± 2.66	2.25 ± 0.95	1.3-6.0
Eosinophils (%)	0 ± 0	0 ± 0	0.5 ± 0.5	0.5 ± 0.29	0.5 ± 0.5	0.5 ± 0.5	0.5-7.2
Basophils (%)	0 ± 0	0 ± 0	0 ± 0	0 ± 0	0 ± 0	0 ± 0	0-0.6
Platelet count (x 10^9/L)	887.25 ± 58.88	1041.5 ± 75.08**	708.5 ± 223.2***###	866.25 ± 62.19 ##	881 ± 126.98 ###	940.5 ± 64.31	529.0-1383.0

Groups	MOR-MET 0.5mg/kg	MOR-MET 1.0mg/kg	MOR-MET 2.0mg/kg	MOR-BUP 0.4mg/kg	MOR-BUP 0.8mg/kg	MOR-BUP 1.6mg/kg	References value
Total RBC (x 10^12/L)	6.85 ± 0.24	7.13 ± 0.14	7.28 ± 0.27	7.13 ± 0.05	6.93 ± 0.25	6.73 ± 0.15	6.39-8.01
Haemoglobin (gm/L)	149 ± 4.4	148.5 ± 3.62	157 ± 4.64	159 ± 4.24	148.75 ± 3.99	145.5 ± 5.87	135-159
PCV (%)	41 ± 1	40 ± 1	43 ± 1	43 ± 1	40 ± 1	40 ± 2	42-49
MCV (fL)	59 ± 1.08	56 ± 0.58	59.25 ± 1.49	59.75 ± 1.03	58.25 ± 1.11	59.25 ± 1.55	58.01-67.00
MCH (pg)	22 ± 0.41	21 ± 0	21.75 ± 0.63	22 ± 0.58	21.75 ± 0.48	22 ± 0.71	18.70-21.20
MCHC (g/L)	370 ± 4.08	372.5 ± 4.79	367.5 ± 2.5	372.5 ± 2.5	372.5 ± 2.5	372.5 ± 2.5	310-336
RDW (%)	15.45 ± 0.45	15.33 ± 0.39	16.65 ± 0.77	15.1 ± 0.17	15.9 ± 0.3	15.95 ± 0.51	13.03-16.57
Total WBC (x 10^9/L)	5.73 ± 1.82	4.13 ± 1.15	4.13 ± 0.27	3.45 ± 0.73	3.83 ± 0.8	4.28 ± 0.21	3.00-9.22
Lymphocytes (%)	58.25 ± 9.59	64.5 ± 1.85	60.75 ± 4.25	65.25 ± 0.25	55.5 ± 7.08	65.75 ± 3.33	51.8-89.7
Monocytes (%)	2.75 ± 1.25	2.5 ± 1.44	3.75 ± 0.85	3 ± 0.58	2.75 ± 0.25	4.5 ± 1.04	1.3-6.0
Eosinophils (%)	0 ± 0	0.25 ± 0.25	0 ± 0	0 ± 0	0.75 ± 0.48	0.25 ± 0.25	0.5-7.2
Basophils (%)	0 ± 0	0 ± 0	0 ± 0	0 ± 0	0 ± 0	0 ± 0	0-0.6
Platelet count (x 10^9/L)	734.25 ± 47.11**###	780.25 ± 80.65###	677.75 ± 57.07***###	670 ± 22.87***###	648.5 ± 60.26***###	843 ± 78.23 ###	529.0-1383.0

Supplementary Table 5: Biochemical analysis on day 5 of all groups of treatment. Means ± SEM of n= 6 rats/group. *P<0.05, **P<0.01, ***P<0.001 vs. Vehicle, #P<0.05, ##P<0.01, ###P<0.001 vs. MOR analysed by two-way repeated measures ANOVA and Bonferroni post-hoc test.

**Substitution Groups**	**VEH**	**MOR-VEH**	**MOR-MG 5mg/kg**	**MOR-MG 10mg/kg**	**MOR-MG 15mg/kg**	**MOR-MG 30mg/kg**	**References value**
Total Bilirubin (umol/L)	1.7 ± 0	1.7 ± 0	1.7 ± 0	1.7 ± 0	1.7 ± 0	1.7 ± 0	0.0-5.1
AST (U/L)	178 ± 22.86	182.25 ± 21.83	165 ± 12.5	150.75 ± 24.21	139.5 ± 7.66	139 ± 7.55	56.1-201.8
ALT (U/L)	65.5 ± 4.87	53.5 ± 0.87	77.5 ± 28.1	57.5 ± 11.51	53.5 ± 5.89	47.5 ± 7.51	34.9-218.1
Alkaline phosphatase (U/L)	275 ± 25.02	285.5 ± 70.12	370.67 ± 69.86 ***##	275 ± 44.08	423.5 ± 102.85***###	433.25 ± 80.69***###	131.6-459.0
Sodium (mmol/L)	145.5 ± 0.96	145.5 ± 1.32	142.5 ± 1.76	144.25 ± 1.11	144.5 ± 1.04	145 ± 0.71	121.9-162.6
Potassium (mmol/L)	8.77 ± 0.45	7.2 ± 0.19	8.45 ± 0.04	8.13 ± 0.56	8.55 ± 0.58	8.58 ± 0.51	4.0-8.0
Chloride (mmol/L)	100.75 ± 0.48	102.25 ± 0.63	104 ± 1.35	103.5 ± 1.19	103.75 ± 0.75	100.75 ± 1.89	81.5-104.0
Urea (mmol/L)	10.25 ± 0.5	6.825 ± 0.94	6.98 ± 1.17	6.55 ± 0.68	6.88 ± 0.54	5.5 ± 0.8	4.32-34.4
Creatinine (umol/L)	55.9 ± 5.51	49.95 ± 2.36	50.63 ± 2.71	43.35 ± 0.97	44.88 ± 1.23	47.08 ± 2.55	35.4-79.6
Total cholesterol (mmol/L)	1.58 ± 0.14	1.23 ± 0.05	1.58 ± 0.22	1.675 ± 0.17	1.5 ± 0.14	1.525 ± 0.11	0.68-1.77
Triglycerides (mmol/L)	0.8 ± 0.15	1.03 ± 0.43	0.98 ± 0.14	0.88 ± 0.25	0.63 ± 0.09	0.55 ± 0.09	0.23-0.99
Calcium (mmol/L)	2.68 ± 0.08	2.5 ± 0.07	2.43 ± 0.06	2.38 ± 0.06	2.5 ± 0.11	2.6 ± 0.07	2.1-2.9
Phosphorus (mmol/L)	3.65 ± 0.16	3.03 ± 0.23	3.3 ± 0.37	2.73 ± 0.28	3.23 ± 0.14	2.98 ± 0.03	1.0-3.94
Total protein (g/l)	68.5 ± 2.33	64 ± 3.34	59.5 ± 2.5	58.75 ± 1.93	62.5 ± 2.96	63.25 ± 0.25	52-71
Albumin (g/l)	30 ± 0.82	26.5 ± 1.04	25.25 ± 0.48	23.25 ± 1.89	27.25 ± 2.25	25.25 ± 1.8	26.85-34.55
Globulin (g/l)	38.5 ± 1.85	37.5 ± 2.99	34.25 ± 2.43	35.5 ± 2.22	35.25 ± 1.25	38 ± 1.78	13-48
A/G Ratio (g/l)	0.775 ± 0.03	0.73 ± 0.05	0.75 ± 0.06	0.65 ± 0.09	0.8 ± 0.06	0.7 ± 0.07	0.6-1.21

Substitution Groups	MOR-BUP 0.4mg/kg	MOR-BUP 0.8mg/kg	MOR-BUP 1.6mg/kg	MOR-MET 0.5mg/kg	MOR-MET 1.0mg/kg	MOR-MET 2.0mg/kg	References value
Total Bilirubin (umol/L)	1.7 ± 0	1.7 ± 0	1.7 ± 0	1.7 ± 0	1.7 ± 0	1.7 ± 0	0.0-5.1
AST (U/L)	173.25 ± 8.38	202 ± 20.99	170.25 ± 18.14	187.25 ± 25.14	191.25 ± 18.63	179.25 ± 13.57	56.1-201.8
ALT (U/L)	58.75 ± 4.31	60.5 ± 10.14	56.25 ± 3.84	53.75 ± 5.82	61.5 ± 9.95	70.25 ± 8.66	34.9-218.1
Alkaline phosphatase (U/L)	274.75 ± 22.25	354.75 ± 60.73**#	294.25 ± 63.25	352.75 ± 57.02**#	313 ± 34.31	313.75 ± 33.4	131.6-459.0
Sodium (mmol/L)	144.5 ± 1.32	143.5 ± 1.44	142.25 ± 0.85	143.25 ± 1.89	143.5 ± 1.5	143.25 ± 0.25	121.9-162.6
Potassium (mmol/L)	8.37 ± 0.53	8.3 ± 1.13	6.45 ± 0.18	7.45 ± 0.53	6.7 ± 0.91	9 ± 0.17	4.0-8.0
Chloride (mmol/L)	102 ± 1.08	103.5 ± 1.19	102.5 ± 1.5	101.25 ± 0.48	101 ± 0.71	102.25 ± 0.25	81.5-104.0
Urea (mmol/L)	9.43 ± 0.51	8.48 ± 0.13	8.25 ± 0.56	8.43 ± 0.97	7.88 ± 0.51	7.75 ± 0.38	4.32-34.4
Creatinine (umol/L)	48.83 ± 2.32	45.33 ± 4.32	45.1 ± 3.09	49.7 ± 6.13	44.68 ± 1.49	49.5 ± 2.32	35.4-79.6
Total cholesterol (mmol/L)	1.73 ± 0.18	1.38 ± 0.12	1.58 ± 0.17	1.43 ± 0.05	1.3 ± 0.04	1.48 ± 0.1	0.68-1.77
Triglycerides (mmol/L)	0.63 ± 0.06	1.13 ± 0.18	0.73 ± 0.05	0.8 ± 0.09	1.13 ± 0.28	0.63 ± 0.13	0.23-0.99
Calcium (mmol/L)	2.53 ± 0.03	2.5 ± 0.07	2.6 ± 0.06	2.55 ± 0.1	2.48 ± 0.08	2.5 ± 0.08	2.1-2.9
Phosphorus (mmol/L)	3.15 ± 0.23	3.05 ± 0.28	2.98 ± 0.2	3.08 ± 0.26	2.65 ± 0.17	2.88 ± 0.1	1.0-3.94
Total protein (g/l)	63.75 ± 1.49	62 ± 2.04	64.75 ± 1.25	62.75 ± 2.29	65.75 ± 1.38	66.5 ± 0.29	52-71
Albumin (g/l)	27.5 ± 0.65	24.5 ± 1.04	26 ± 1.73	25.75 ± 2.36	26.75 ± 0.75	27.25 ± 1.44	26.85-34.55
Globulin (g/l)	36.25 ± 1.44	37.5 ± 1.66	38.75 ± 1.44	37 ± 0.91	39 ± 1.22	39.25 ± 1.6	13-48
A/G Ratio (g/l)	0.8 ± 0.04	0.68 ± 0.03	0.68 ± 0.06	0.68 ± 0.06	0.7 ± 0.04	0.7 ± 0.07	0.6-1.21

Supplementary Table 6: The microscopic structures of the lung; pulmonary vessel (PV), bronchiole (B), alveoli (AV); heart; mycocyte (n), myocardiums (M), blood vessels (BV); liver; central vein (CV), sinusoids (S), endothelial cells (EC), hepatocytes (H) and kidney; glomerulus (G), Bowman's capsule (BC), renal corpuscle (RC).

Groups/ Organ	Heart	Lung	Kidney	Liver
VEH	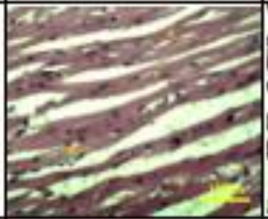	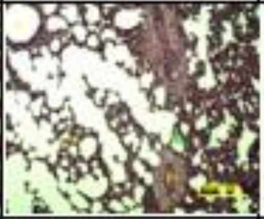	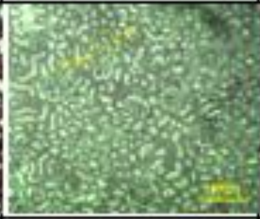	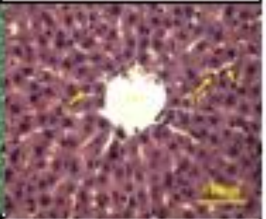
MOR	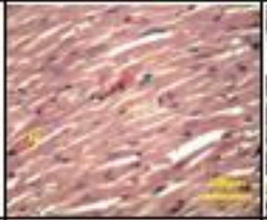	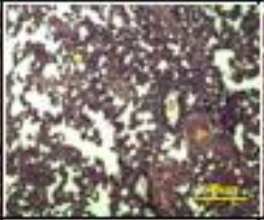	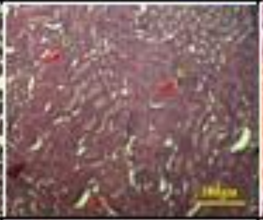	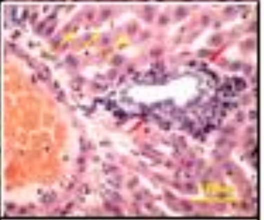
MOR - MG 5 MG/KG	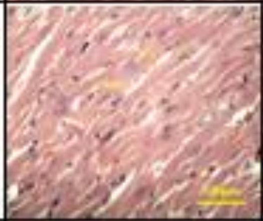	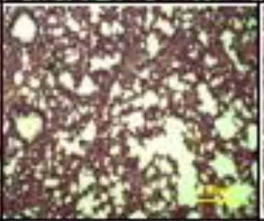	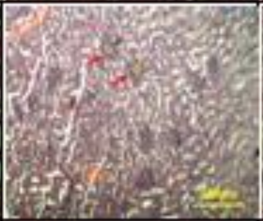	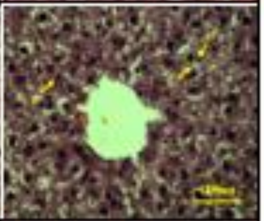
MOR - MG 30 MG/KG	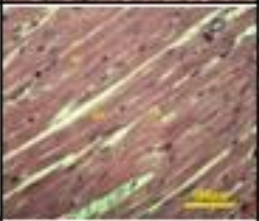	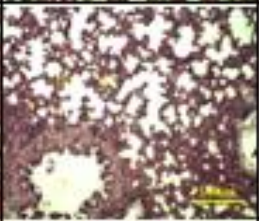	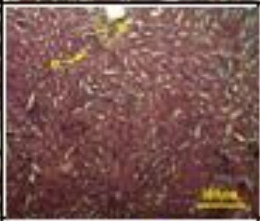	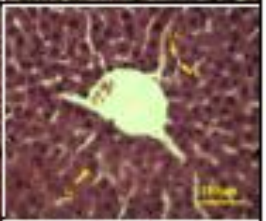
MOR - MET 1 MG/KG	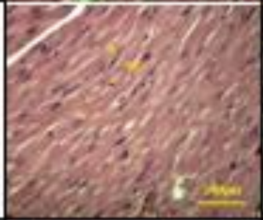	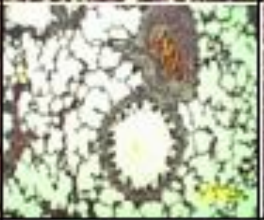	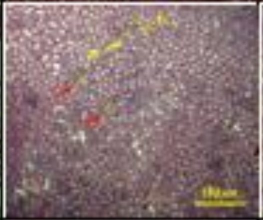	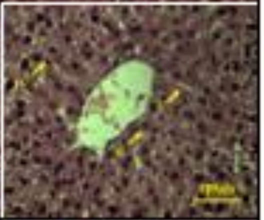
MOR - BUP 0.8 MG/KG	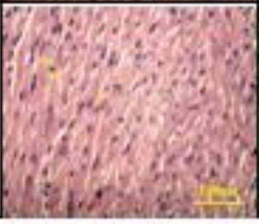	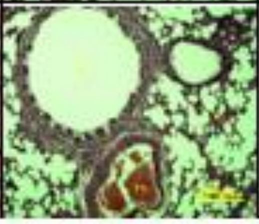	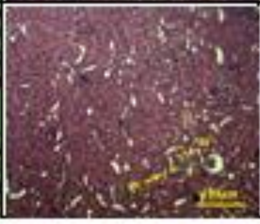	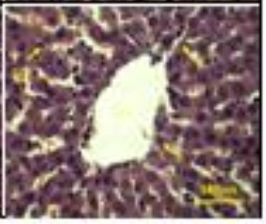
